# Mix and match backbones for the formation of H-bonded duplexes†
†Electronic supplementary information (ESI) available: Detailed experimental procedures with spectroscopic characterization data, ^31^P NMR titration spectra and binding isotherms. See DOI: 10.1039/c5sc04467g



**DOI:** 10.1039/c5sc04467g

**Published:** 2016-01-07

**Authors:** Giulia Iadevaia, Alexander E. Stross, Anja Neumann, Christopher A. Hunter

**Affiliations:** a Department of Chemistry , University of Cambridge , Lensfield Road , Cambridge CB2 1EW , UK . Email: herchelsmith.orgchem@ch.cam.ac.uk; b Department of Chemistry , University of Sheffield , Sheffield S3 7HF , UK

## Abstract

The formation of well-defined supramolecular assemblies involves competition between intermolecular and intramolecular interactions, which is quantified by effective molarity. Formation of a duplex between two oligomers equipped with recognition sites displayed along a non-interacting backbone requires that once one intermolecular interaction has been formed, all subsequent interactions take place in an intramolecular sense. The efficiency of this process is governed by the geometric complementarity and conformational flexibility of the backbone linking the recognition sites. Here we report a series of phosphine oxide H-bond acceptor AA 2-mers and phenol H-bond donor DD 2-mers, where the two recognition sites are connected by isomeric backbone modules that vary in geometry and flexibility. All AA and DD combinations form stable AA·DD duplexes, where two cooperative H-bonds lead to an increase in stability of an order of magnitude compared with the corresponding A·D complexes that can only form one H-bond. For all six possible backbone combinations, the effective molarity for duplex formation is approximately constant (7–20 mM). Thus strict complementarity and high degrees of preorganisation are not required for efficient supramolecular assembly. Provided there is some flexibility, quite different backbone modules can be used interchangeably to construct stable H-bonded duplexes.

## Abstract 2

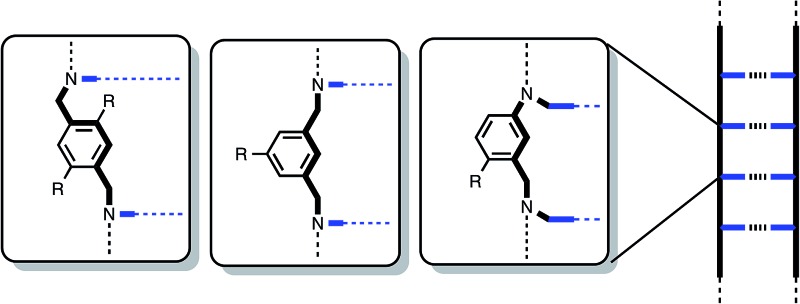
Six isomeric backbone combinations can be used interchangeably to construct stable H-bonded duplexes of similar stability.

## Introduction

Linear polymers equipped with complementary recognition sites have the potential to reproduce the functional properties of nucleic acids: sequence selective duplex formation, templated synthesis, self-replication and forced evolution.^1^ Modified versions of DNA have been prepared, where the phosphate linker,^2,3^ the sugar,^4^ the backbone or the base pairing system^5^ have been replaced, and these systems all form stable duplexes.^6^ The success of these systems suggests that it might be possible to make completely different classes of synthetic information molecule that bear no resemblance to their biological counterparts.

**Fig. 1:**
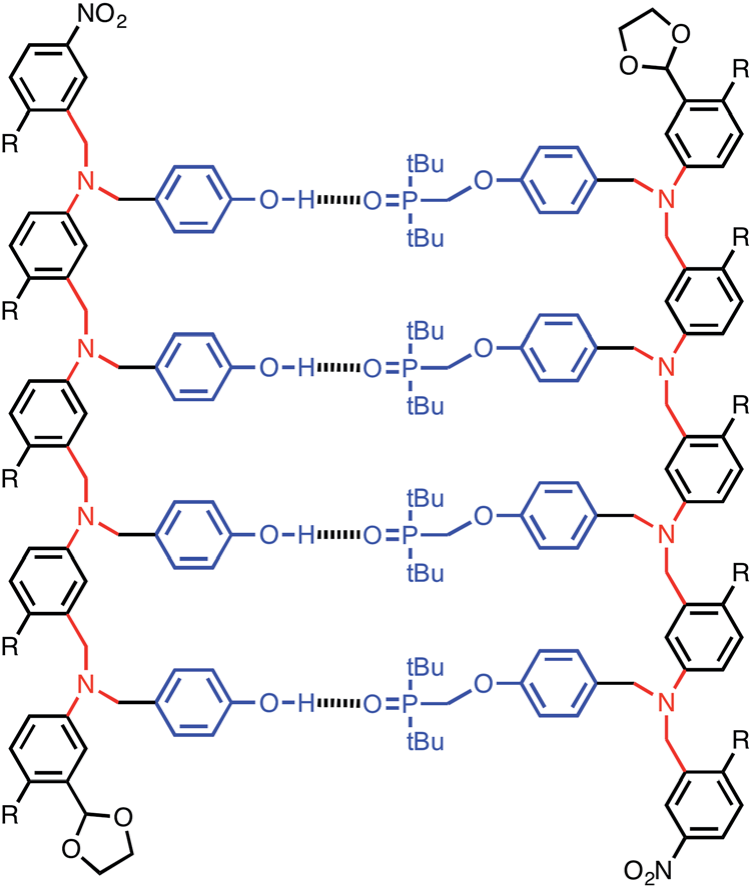
The duplex formed by a phenol 4-mer (DDDD) and a phosphine oxide 4-mer (AAAA). R is a 2-ethylhexoxy group that provides solubility in toluene (the anti-parallel structure is shown, but the parallel structure is also possible).

A number of synthetic systems that form duplexes *via* different non covalent interactions have been reported.^7^ Lehn described oligo(2,2′-bipyridine) ligands that self-assemble into length specific helical duplexes in the presence of a metal ion.^7*a*^ Lehn also reported ligands containing sequences of bidentate (bipyridine) and tridentate (terpyridine) binding sites which form sequence specific helicates depending on the properties of the metal directing the assembly.^7*b*^ Huc and Lehn described pyridinecarboxamide oligomers that form double helices due to aromatic stacking interactions.^7c,d^ Anderson synthesized zinc porphyrin oligomers that assemble into ladders in the presence of 1,4-diazabicyclo[2.2.2]octane (DABCO). A linear increase in stability with the length of the ladder was observed, which indicates cooperative duplex formation, and when oligomers of different lengths were mixed in the presence of DABCO, only length complementary ladders were formed.^7e,f^ deMendoza described guanidinium oligomers that form double helices held together by H-bonding interactions with the sulfate counterions.^7*g*^ Hunter reported oligoamides that form duplexes *via* H-bonding and edge-to-face aromatic interactions. An increase in stability with increasing length was observed indicating cooperative assembly.^7h,i^ Gong reported oligoamides containing different sequences of H-bond donor and H-bond acceptor sites that show sequence selective duplex formation.^7j–o^ Chen reported self-complementary oligomeric hydrazide oligomers and amidourea oligomers both of which form H-bonded duplexes.^7p–r^ Different length spacers between the H-bonding sites were used to obtain selective assembly of complementary oligomers. Krische reported aminotriazine and diaminopyridazine oligomers that form H-bonded duplexes.^7s,t^ Yashima described oligomers that form helical duplexes through salt bridge interactions between amidinium and carboxylate sites.^7u–w^ These systems show sequence selective and length selective duplex formation, and a template strand bearing two amidinium binding sites was used to direct the synthesis of a complementary strand bearing two carboxylate units.^7*x*^ We recently reported a new class of linear oligomeric molecules that form stable duplexes *via* the formation of multiple cooperative H-bonding interactions (Fig. 1).^8^ Specifically, oligomers equipped with phenol H-bond donors formed 1 : 1 complexes with oligomers equipped with phosphine oxide H-bond acceptors. The stability of the duplex increases by of an order of magnitude for every additional H-bond formed.

**Fig. 2:**
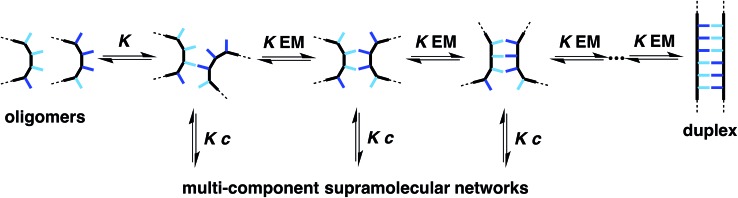
Stepwise assembly of a duplex from two complementary oligomers. There is an intermolecular channel that leads to cross-linked polymeric networks and an intramolecular channel that leads to duplex formation. *K* is the association constant for formation of an intermolecular interaction between two complementary H-bonding sites (blue bars), EM is the effective molarity for formation of an intramolecular interaction, and *c* is the operating concentration.

The efficiency of duplex formation is determined by the stepwise equilibria shown in Fig. 2.^9^ The first interaction in duplex assembly is an intermolecular H-bond which has an association constant *K* (≈300 M^–1^ for the phosphine oxide–phenol H-bond). The second H-bond is an intramolecular interaction with an equilibrium constant *K* EM, where EM is the effective molarity for the intramolecular process (≈10 mM for the duplex in Fig. 1). All subsequent H-bonds are intramolecular and were found to have similar effective molarities for the system in Fig. 1. Duplex formation requires that *K* EM ≫ 1 to ensure that once the first intermolecular H-bond is made, all subsequent intramolecular H-bonds are highly favoured, and there is no competition from intermolecular interactions that would lead to uncontrolled aggregation (intermolecular channel in Fig. 2). For the system shown in Fig. 1, *K* EM ≈ 5, so duplex formation is reasonably efficient, but there are partially bound states present where the H-bonding interactions are broken some of the time. There are two strategies to improve duplex formation: increase *K* by changing the H-bonding groups, or increase EM by changing the supramolecular architecture. In this paper, we explore the effect of backbone architecture on the effective molarity for duplex formation.

**Fig. 3:**
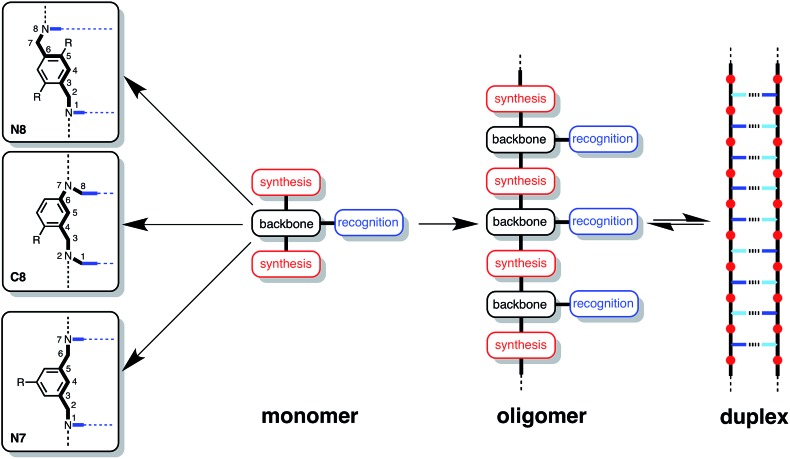
A blueprint for duplex forming molecules. There are three key design elements: one module which defines the chemistry for the synthesis of oligomers (red), the recognition module which controls intermolecular binding (blue) and the backbone module which links these components together. Three isomeric backbone modules are shown (R are sites for attachment of solubilising groups). Adapted from ref. 8.

The design of the oligomer architecture in Fig. 1 is modular. Fig. 3 illustrates the basic blueprint. The recognition modules (blue), the chemistry used to synthesise oligomers (red) and the backbone (black) have been incorporated into the chemical structure in Fig. 1, such that different properties of the system can be independently varied, *i.e.* one module can be changed without affecting the other two. Fig. 3 illustrates how the backbone can be changed, but keeping the reductive amination chemistry that was used for synthesis of the compounds in Fig. 1 and the same phosphine oxide–phenol recognition module. Here we investigate changing the constitution of the backbone by measuring the effect of the three isomeric backbone modules in Fig. 3 on the EM for duplex formation for different combinations of AA and DD 2-mers. Each backbone module is each composed of an aromatic ring and two methylene groups, but differences in connectivity leads to a variation in the geometry and flexibility of the motif linking the recognition modules. The nomenclature N8/C8/N7 indicates the atom to which the recognition module is attached and the length of the linker. The C8 backbone has three flexible methylene groups connecting the recognition sites, while the N8 and N7 backbones each have two. Thus the N8 backbone is relatively rigid and extended, the N7 backbone is rigid and shorter, and the C8 backbone is flexible, so that it can explore both elongated and compact conformations.

## Results and discussion

### Synthesis

**Scheme 1:**
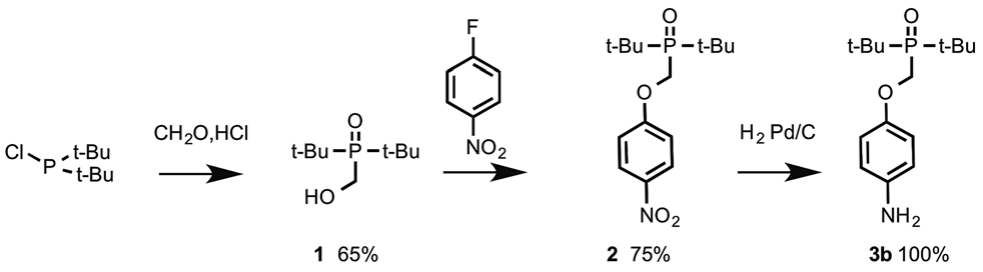


The N8 and N7 backbones were accessed by reductive amination of dialdehydes with anilines bearing the recognition groups. The H-bond donor aniline 4-aminophenol is commercially available. The H-bond acceptor aniline bearing a phosphine oxide group was synthesized as shown in Scheme 1. Di-*t*-butyl(chloro)phosphane was treated with formaldehyde to give alcohol **1**, which was reacted with 4-fluoro-nitrobenzene in the presence of K_2_CO_3_ to give **2**.^10^ Reduction of **2** gave aniline **3b**.

**Scheme 2:**
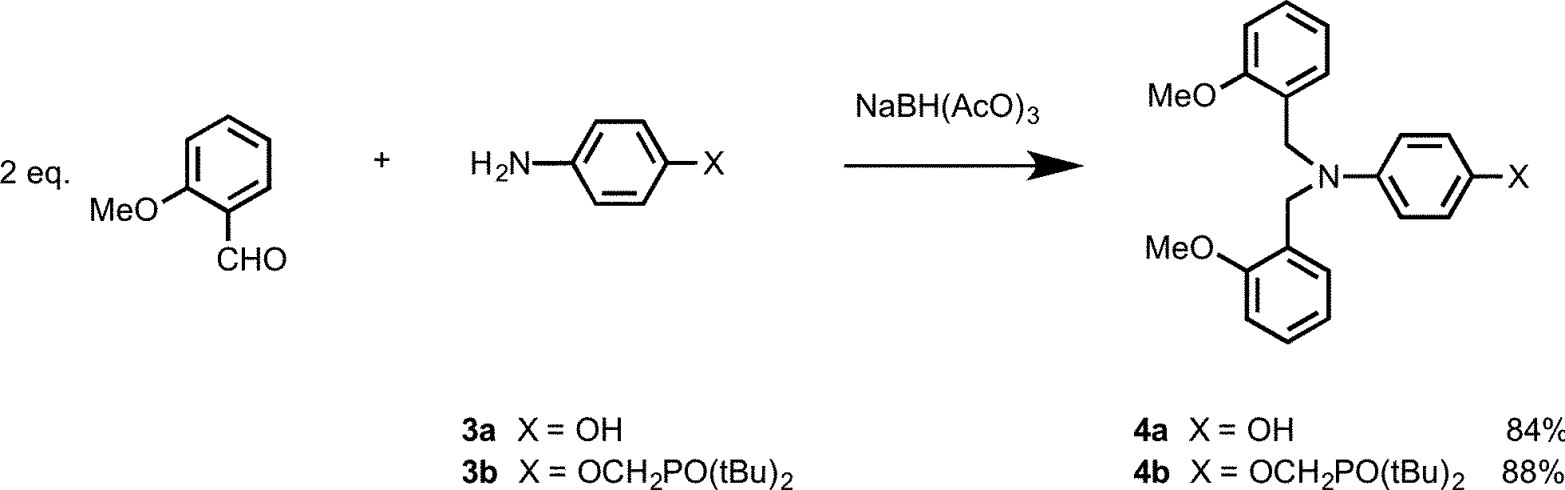


In order to determine effective molarities, H-bond acceptor and donor 1-mers are required to measure the intermolecular association constant *K*. The H-bond donor parameter of 3-dimethylaminophenol is somewhat lower than the value for phenol (*α* = 3.5 compared with 3.8), which suggests that there may be a substituent effect on the strengths of H-bonding interactions involving donors attached to the C8 backbone compared with donors attached to the N7 and N8 backbones.^11^ The 1-mers corresponding to the N7 and N8 backbones, **4a** and **4b** (A and D), were therefore synthesized from **3a** or **3b** by reductive amination with 2-methoxybenzaldehyde (Scheme 2).^12^

**Scheme 3:**
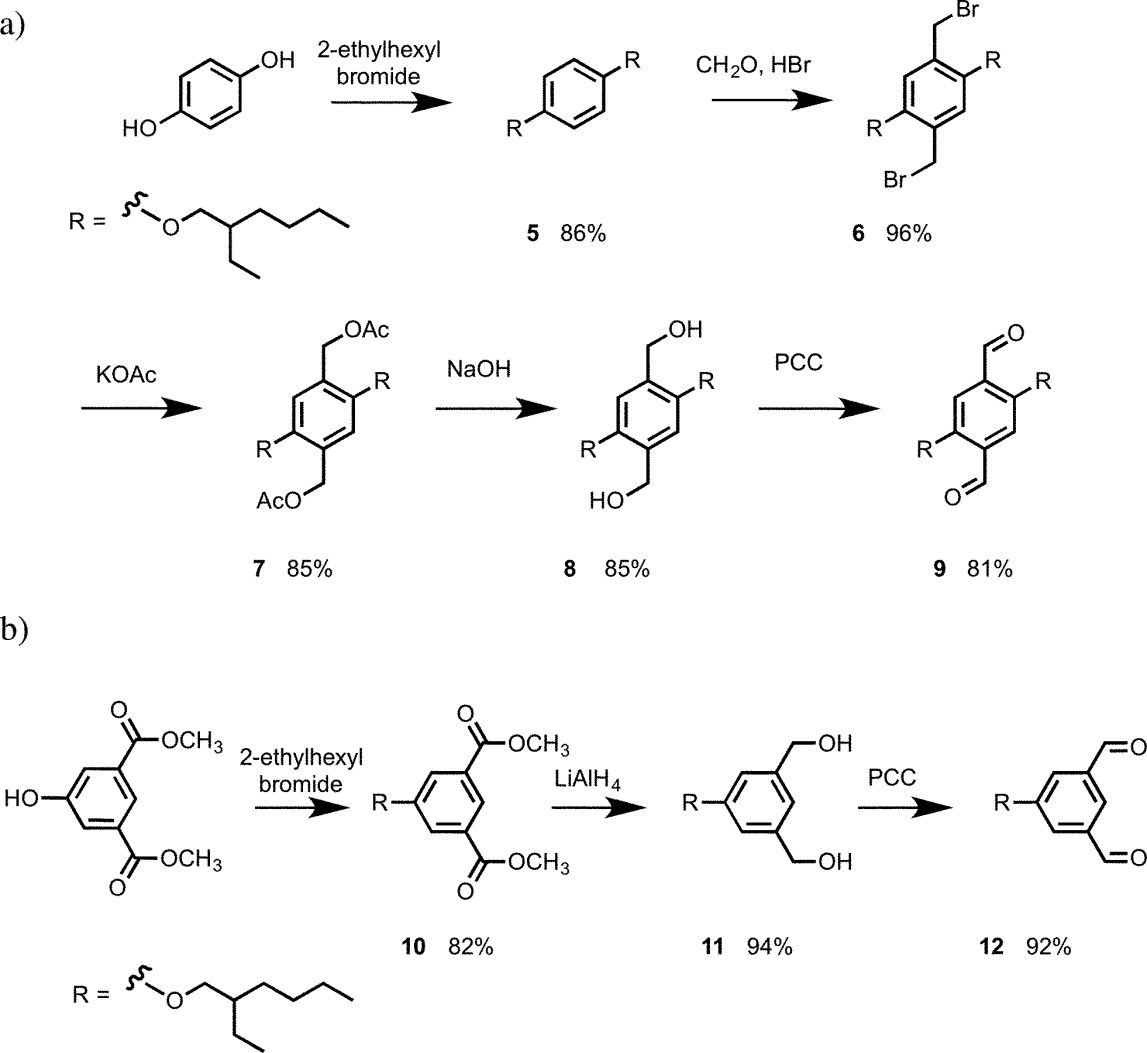


The 1,4-dialdehyde **9** required for the N8 backbone was synthesised using a sequence of bromomethylation–acetylation–reduction–oxidation reactions as shown in Scheme 3.^13^ The 1,3-dialdehdye **12** required for the N7 backbone was prepared from dimethyl 5-hydroxyisophthalate: alkylation of the phenol group with 2-ethylhexylbromide, followed by reduction of the esters with LiAlH_4_ gave diol **11**, and oxidation of **11** with PCC gave **12** (Scheme 3).

**Scheme 4:**
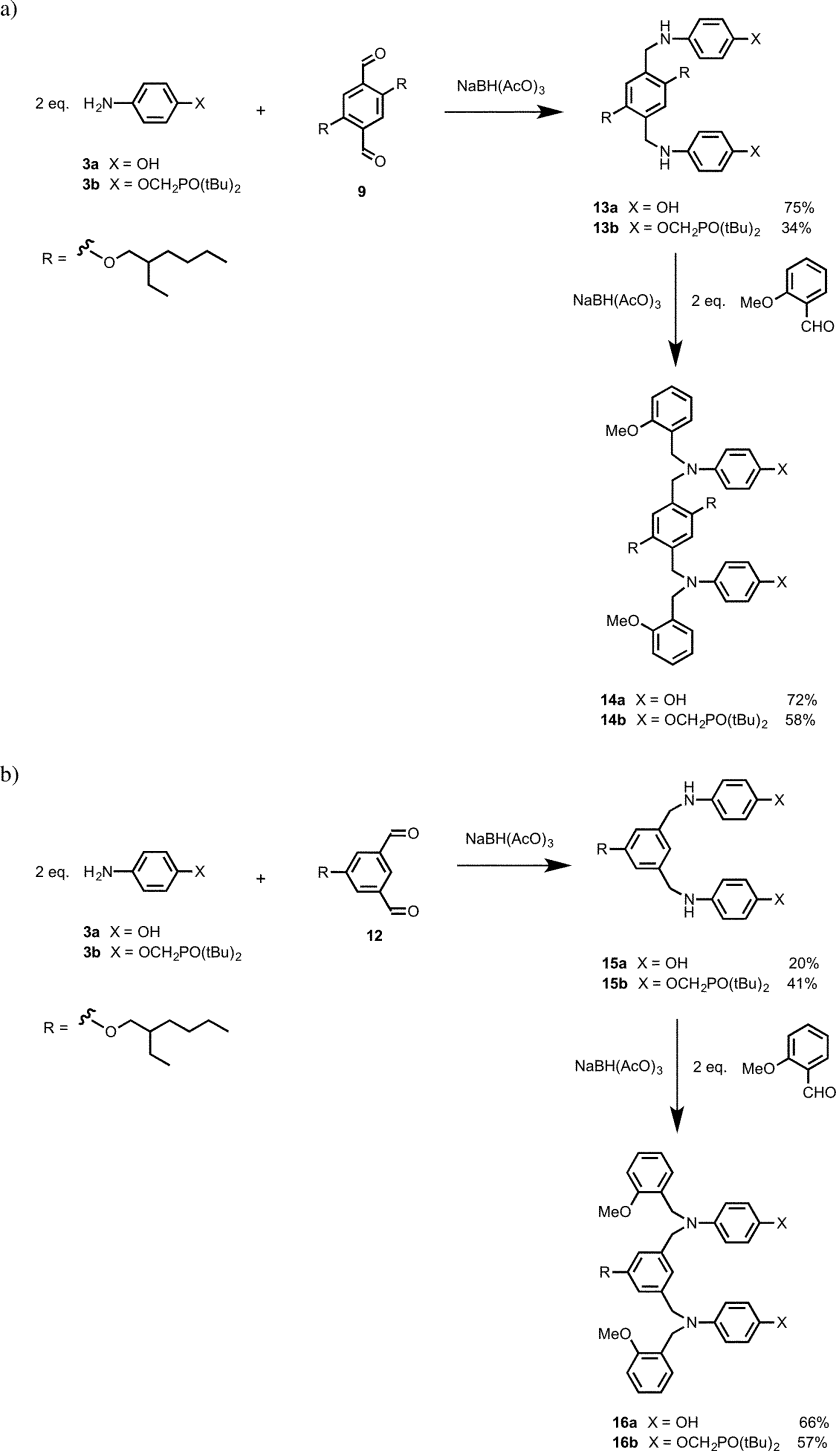


Compounds **13a** and **13b** were synthesized by reacting two equivalents of anilines **3a** or **3b** with dialdehyde **9** (Scheme 4a). A further reductive amination step was used to cap **13a** and **13b** with 2-methoxybenzaldehyde to give **14a** and **14b**, the N8 backbone DD and AA 2-mers. Similarly **15a** was synthesized by reductive amination of dialdehyde **12** with aniline **3a** (Scheme 4b). Compound **15b** was obtained by reacting dialdehyde **12** and two equivalents of aniline **3b** to obtain the diimine, which was then reduced with NaBH_4_ to give **15b**. Compounds **15a** and **15b** were then capped by with 2-methoxybenzaldehyde under reductive amination conditions to obtain **16a** and **16b**, the N7 backbone DD and AA 2-mers (Scheme 4b).

**Fig. 4:**
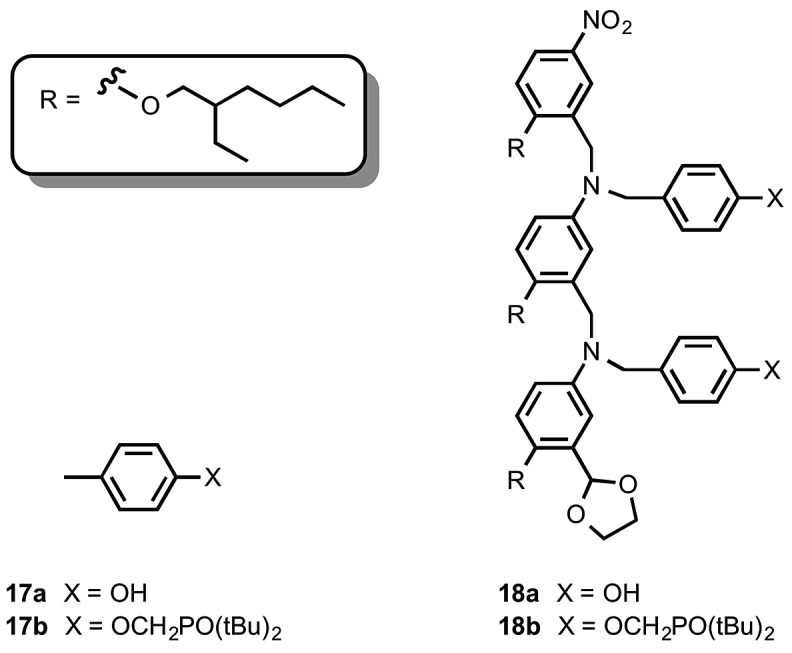
C8 backbone 1-mers (A and D) and 2-mers (AA and DD).^8^

The C8 backbone AA and DD 2-mers belong to the family of oligomers illustrated by the AAAA and DDDD 4-mers in Fig. 1. The synthesis of these compounds (**18a** and **18b**) and the corresponding 1-mers (**17a** and **17b**) used to determine effective molarities was reported previously (Fig. 4).^8^

### Binding studies

Binding studies for pairwise combinations of the AA and DD 2-mers were carried out by means of ^31^P NMR titrations in toluene. The corresponding 1-mers, A and D, were used to measure the strength of a single intermolecular phenol–phosphine oxide H-bond. The H-bond acceptor phosphine oxides were used as the host, and a large increase in the ^31^P NMR chemical shift was observed upon guest addition in all cases, which is indicative of H-bond formation.^14^ The titration data were fit to a 1 : 1 binding isotherm to obtain the association constants and limiting complexation-induced changes in chemical shift (Table 1). The association constants for the two A·D complexes are similar, which indicates that substituent effects on the phenol–phosphine oxide H-bond are not significant in these systems. The association constants for the AA·DD complexes are significantly larger than the values for the corresponding A·D complexes in all cases, which implies that two H-bonds are formed in a cooperative manner in the 2-mer duplexes. The large limiting complexation-induced changes in ^31^P NMR chemical shift, Δ*δ*, observed for the AA·DD complexes support this conclusion. Effective molarities for the formation of the second intramolecular H-bond in the AA·DD complexes were determined using eqn (1), and the values are reported in Table 1.1
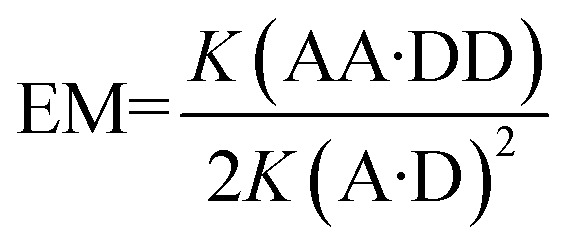



**Table 1:** Association constants (*K*), effective molarities (EM) and limiting complexation-induced changes in chemical shift obtained by fitting ^31^P titration data in toluene at 298 K to a 1 : 1 binding isotherm

Complexes	Backbone	*K*/M^–1^	Δ*δ* ^31^P/ppm	EM/mM	*K* EM
**A·D**					
**4a·4b**		250 ± 10	5.0		
**17a·17b** ^8^		350 ± 20	4.9		

**AA·DD**					
**14a·14b**	N8·N8	2500 ± 200	4.0	20 ± 2	5 ± 1
**16a·16b**	N7·N7	1200 ± 400	6.9	10 ± 3	2 ± 1
**18a·18b** ^8^	C8·C8	1900 ± 600	5.3	8 ± 3	3 ± 1
**16a·14b**	N7·N8	900 ± 200	6.6	7 ± 2	2 ± 1
**14a·18b**	N8·C8	1400 ± 200	3.7	11 ± 2	3 ± 1
**16a·18b**	N7·C8	920 ± 60	6.0	7 ± 1	2 ± 1

**Fig. 5:**
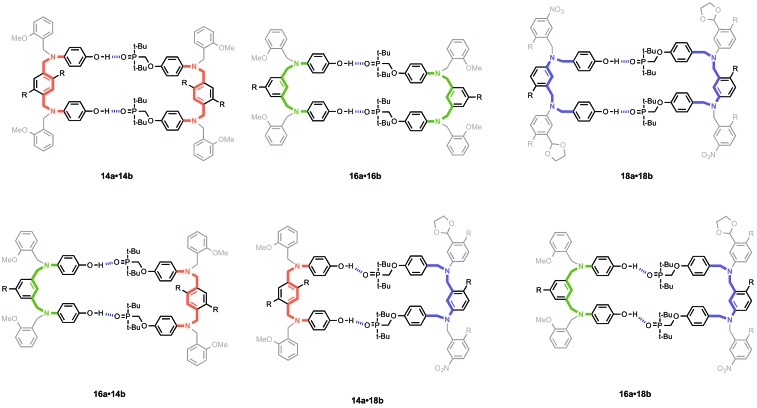
Six different backbone combinations that lead to equally stable duplexes. The C8 backbone is highlighted in purple, the N8 backbone in orange, and the N7 backbone in green. The anti-parallel structure of **18a·18b** is shown, but the parallel structure is also possible.

The effective molarities are very similar for all backbone combinations, indicating that the ability to form a duplex does not depend strongly on the conformational properties of the backbone in these systems. The values of EM in Table 1 are consistent with the values found for other supramolecular systems which generally fall in the window 10–1000 mM.^15,16^ Fig. 5 illustrates the six different backbone combinations investigated in this paper. The backbones units all have two methylene groups, and it seems that these linkages provide sufficient flexibility to allow the backbones to adapt to quite different geometrical requirements. The outcome is that any backbone combination leads to stable duplex formation, and the duplex architecture illustrated in Fig. 3 is truly modular with respect to the backbone component.

**Fig. 6:**
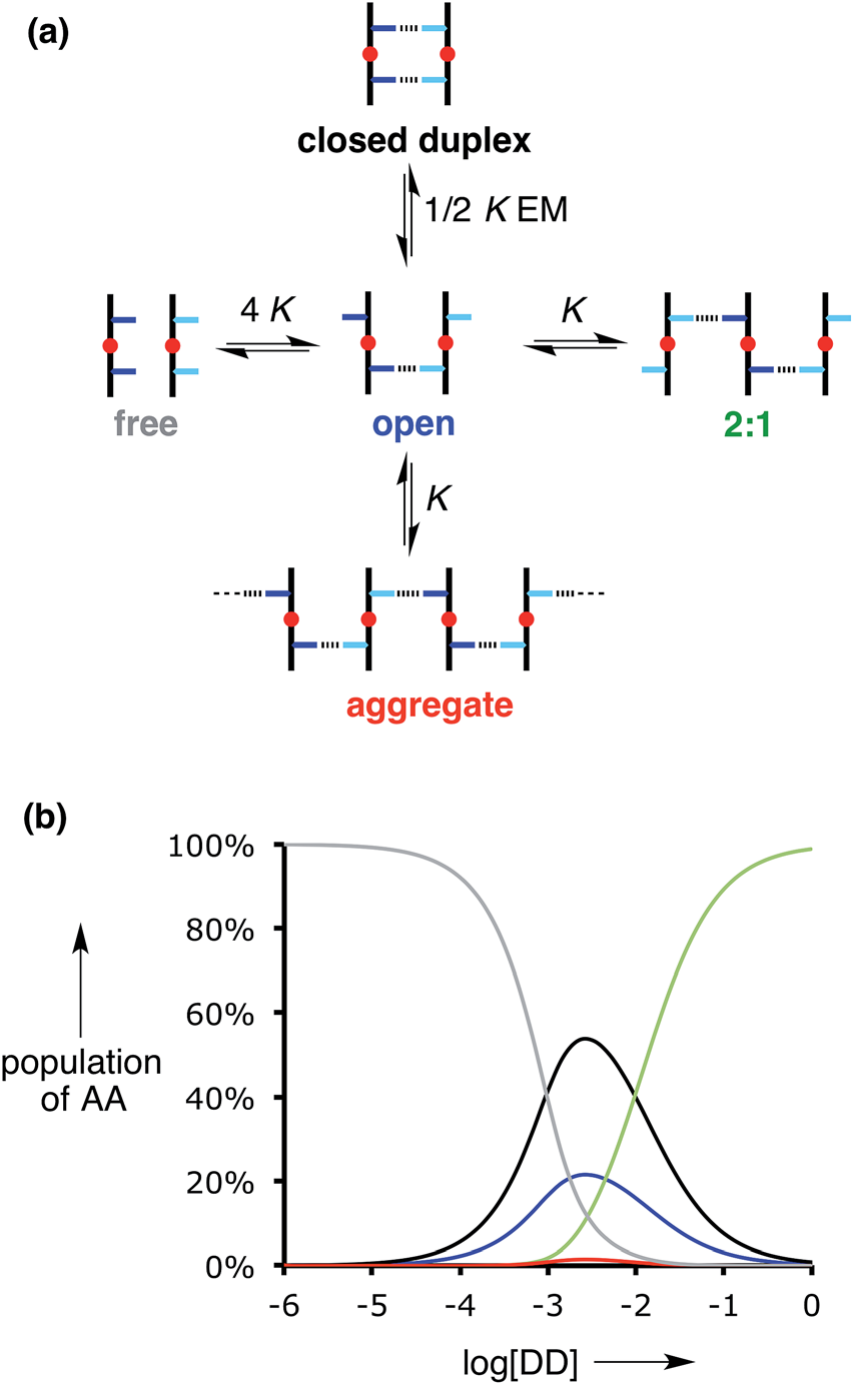
(a) Complexes that compete with duplex formation. There are three species with a 1 : 1 stoichiometry: the closed doubly H-bonded complex, the open singly H-bonded complex, and the open polymeric aggregate. When one component is present in excess, the 2 : 1 complex is also possible. (b) Speciation of AA as a function of the concentration of DD ([AA] = 1 mM, *K* = 300 M^–1^ and *K* EM = 5). Free unbound AA is shown in grey, the fully bound AA·DD duplex in black, the partially bound AA·DD duplex in blue, polymeric aggregates in red, and the 2 : 1 AA·DD_2_ complex in green.

Table 1 also lists the values of *K* EM for the six different backbone combinations. The values are similar for all of the duplexes, and in all cases *K* EM is greater than one, indicating that intramolecular H-bonding is favoured. However, the values of *K* EM are not very much greater than one, which implies that the partially bound open complex shown in Fig. 6a is populated to a significant extent. The open complex could also aggregate, and the other species that competes with formation of the closed doubly H-bonded 1 : 1 duplex is formation of a 2 : 1 complex (Fig. 6a). Fig. 6b illustrates the speciation of AA for titration of DD into 1 mM AA for *K* EM = 5. Under these conditions, the population of polymeric aggregate (red) is negligible, and this will always be the case when the concentration of one of the oligomers is less than 1/*K* (≈3 mM). The duplex (black) is the major species present in a 1 : 1 mixture of AA and DD (≈50%). The partially bound open complex (blue) is also significantly populated (≈20%), and the 2 : 1 AA·DD_2_ complex (green) dominates in the presence of excess DD. The titration data were also analysed using a 2 : 1 binding isotherm to allow for the formation of this species towards the end of the titration, but the association constants determined for the 1 : 1 complexes were not significantly affected.

There are important implications of the results in Table 1 and the speciation diagram in Fig. 6 for the formation of longer duplexes from the building blocks described in this paper. As the lengths of the oligomers increase, the stabilities of the fully assembled duplexes will increase in proportion to (*K* EM)^*N*^, where *N* is the number of recognition modules. Although, the number of possible competing complexes will increase with *N*, the stabilities of these complexes will not increase proportionately, so off-pathway complexes will become increasingly less significant as *N* increases. Fig. 6b shows that even for *N* = 2, the values of *K* EM are sufficiently high that intermolecular processes leading to the formation of higher order complexes or polymeric aggregates do not compete with duplex formation at mM concentrations (<3% in total for a 1 mM mixture of AA and DD compared with 58% of the two 1 : 1 complexes). On the other hand, partially bound states of the duplex will be significantly populated for longer oligomers. Unless there is cooperative coupling of neighbouring H-bonding interactions along a duplex, the probability of breaking a H-bond will be independent of *N* and proportional to (*K* EM)^–1^. Previous results on the C8 backbone oligomers confirm that the C8·C8 combination with *K* EM = 3 is capable of propagating the assembly longer duplexes (*e.g.* the AAAA·DDDD complex illustrated in Fig. 1) without competition from higher order aggregates. The results presented here suggest that any of the three different backbones can be used interchangeably to construct stable H-bonded duplexes.

## Conclusions

We previously reported a modular strategy for the construction of synthetic information molecules, where complementary H-bonding sites are displayed along a non-polar backbone. In this paper, we investigate how well different backbone modules are tolerated by investigating effect of isomeric linkers on the recognition properties of H-bond donor and acceptor 2-mers (DD and AA). Three different phosphine oxide AA 2-mers and three different phenol DD 2-mers were synthesised using linkers that vary in geometry and conformational flexibility. NMR titrations were used to characterise the AA·DD duplexes formed by all of the six possible backbone combinations. The association constants for formation of the AA·DD complexes (10^3^ M^–1^) are all an order of magnitude higher than the association constants for formation of the corresponding A·D complexes (10^2^ M^–1^), which can only form one H-bond. In addition, the complexation-induced changes in ^31^P NMR chemical shift are indicative of fully H-bonded complexes in all cases, indicating that all six backbone combinations lead to duplex formation with cooperative formation of two H-bonds. The values of EM measured for intramolecular H-bond formation leading to duplex formation are remarkably insensitive to the nature of the backbone (7–20 mM). Thus any of the backbone modules described in this paper could be used interchangeably to construct stable H-bonded duplexes of longer oligomers. There is no strong dependence of EM on geometric complementarity or conformational flexibility. It seems that provided the backbone has sufficient flexibility to allow the H-bonding sites to connect, the precise choice of linker is not critical.
