# Supplementary material for: Mix and match backbones for the formation of H-bonded duplexes
Source: Chem Sci. 2016 Jan 7;7(3):1760–7. doi: 10.1039/c5sc04467g (PMC5592378; doi:10.1039/c5sc04467g)
Supplement: Supplementary file 1 [file SC-007-C5SC04467G-s001.pdf]

Electronic Supplementary Material (ESI) for Chemical Science.
This journal is © The Royal Society of Chemistry 2015

Mix and Match Backbones for the Formation of H-Bonded Duplexes

Giulia Iadevaia,[a] Alexander E. Stross,[a] Anja Neumann,[b] Christopher A. Hunter [a,*]

[a] Department of Chemistry, University of Cambridge, Lensfield Road, Cambridge CB2 1EW (UK)

[b] Department of Chemistry, University of Sheffield, Sheffield S3 7HF (UK)

Supplementary Information

Synthesis

Synthesis of 1

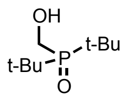

To 80 ml 37% aqueous formaldehyde and 80 ml concentrated HCl solution, di-tert-butyl(chloro)phosphane (8.2 ml, 43.0 mol) was added and the mixture was heated to 100˚C and stirred overnight. The solution was allowed to cool, then neutralized with NaOH (14 g) and $NaHCO_3$ (7.0 g), extracted with $CHCl_3$ (200 x 3 ml), washed with brine (100 ml), dried with Na_2SO_4 and the solvent was removed under reduced pressure. The crude was then purified by recrystallization with hexane at 69˚C. The product was isolated as a white solid (5.4 g, 65%).

^{1}H NMR (250 MHz, $CDCl_3$): δ 4.04 (s, 2H), 1.28 (d, J = 13, 18H);
^{31}P NMR (101.2 MHz, $CDCl_3$): δ 58.7;
^{13}C NMR (62.9 MHz, $CDCl_3$): δ 55.2, 54.3, 35.3, 34.5, 26.5;
MS (ES+): m/z (%) = 193.1 (100) [$M+H^+$];
HRMS (ES+): calcd for $C_9H_{22}O_2P$ 193.1257, found 193.1348;
FT-IR (thin film): ν_{max} /cm^{-1} 3148, 2871, 2954;
m.p.: 149-150 °C.

Synthesis of 2 [1]

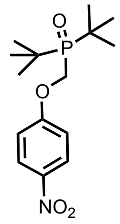

A mixture of 4-fluoronitrobenzene (0.4 g, 2.6 mmol), **1** (1.0 g, 5.2 mmol) and Cs_2CO_3 (1.7 g, 5.2 mmol) in DMF (10 ml) was heated at 100°C for 12 h. After cooling to room temperature, the solution was diluted with EtOAc (100 ml), washed with water (3 x 100 mL) and brine (1 x 100 ml), dried with $MgSO_4$, and the solvent was removed under reduced pressure. The crude material was isolated as an orange solid and used without further purification (0.62 g, 75%)

^{1}H NMR (400 MHz, $CDCl_3$**):** δ 8.26 (d, J = 9, 2H), 7.05 (d, J = 9, 2H), 4.49 (d, J = 7, 2H), 1.41 (d, J = 14, 18H);
^{31}P NMR (162.0 MHz, aceton-d_6): δ 56.1;
^{13}C NMR (100.6 MHz, aceton-d_6): δ 163.1, 163.0, 142.5, 126.1, 114.4, 63.5, 62.8, 35.9, 35.3, 26.5;
MS (ES+): m/z (%) = 314.1 (100) [$M+H^+$];
HRMS (ES+): calcd for $C_{15}H_{25}NO_4P$ 314.1521 found 314.1524;
FT-IR (thin film): ν_{max} /cm^{-1} 3407, 2966, 2911, 2876, 1608, 1602, 1592, 1513, 1503.
m.p.: 150-162 °C

Synthesis of 3b

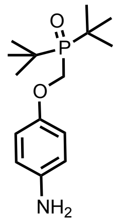

A mixture of **2** (0.3 g, 0.95 mmol) and palladium (10% activated on carbon) (0.02 g) in EtOAc (5 ml) was stirred at room temperature under one atmosphere of hydrogen, for 12 h. The mixture was filtered through silica and the filtrate was evaporated to give pure product as an orange solid (0.28 g, 100%).

^{1}H NMR (250 MHz, $CDCl_3$): δ 6.81-6.70 (m, 4H), 4.34 (d, J = 7, 2H), 1.39 (d, J = 14, 18H);
^{31}P NMR (101.2 MHz, CD_3CN): δ 55.2;
^{13}C NMR (100.6 MHz, $CDCl_3$): δ 152.0, 151.9, 140.0, 116.9, 115.1, 63.5, 62.8, 35.7, 35.1, 26.5;
MS (ES+): m/z (%) = 284.2 (100) [M+H^+];
HRMS (ES+): calcd for $C_{15}H_{27}NO_2P$ 284.1779, found 284.1771;
FT-IR (thin film): ν_{max} /cm- 1 3399, 3325, 3218, 2954, 2903, 2870, 1510;
m.p.: 139-141 °C.

Synthesis of 4a

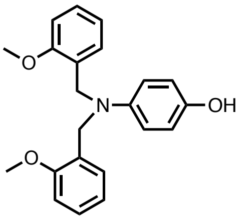

A mixture of **3a** (1.0 g, 9.2 mmol), 2-methoxybenzaldehyde (2.8 ml, 23 mmol) and $NaBH(AcO)_3$ (5.8 g, 27 mmol) in DCE (60 ml) dried with molecular sieves, was stirred under nitrogen at room temperature for 3 h. The solution was then washed with saturated aqueous $NaHCO_3$ (1 x 100 ml), water (1 x 100 ml) and brine (1 x 100 ml), dried with $MgSO_4$, and the solvent was removed under reduced pressure. The crude product was then purified by column chromatography on silica eluting with hexane/EtOAc (90:10). The product was isolated as a brown solid (2.7 g, 84%).

^{1}H NMR (500 MHz, CD_3CN): δ 7.21 (td, J = 8, J = 1, 2H), 7.13 (d, J = 7, 2H), 6.95 (d, J = 8, 2H), 6.85 (td, J = 7, J = 1, 2H), 6.57 (d, J = 8, 2H), 6.47 (d, J = 8, 2H), 6.14 (s, 1H), 4.52 (s, 4H), 3.81 (s, 6H);
^{13}C NMR (125.7 MHz, CD_3CN): δ 158.4, 148.9, 143.8, 128.8, 128.4, 127.9, 121.2, 116.7, 114.5, 111.4, 56.0, 51.0;
MS (ES+): m/z (%) = 350.2 (100) [$M+H^+$];
HRMS (ES+): calcd for $C_{22}H_{24}NO_3$ 350.1756, found 350.1759;
FT-IR (thin film): ν_{max} /cm^{-1} 3389, 3035, 2940, 2845, 1593, 1601, 1514;
m.p.: 116-118 °C.

Synthesis of 4b [2]

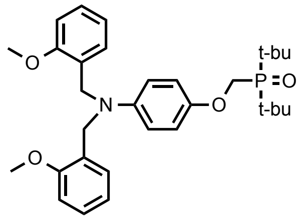

A mixture of **3b** (0.06 g, 0.21 mmol), 2-methoxybenzaldehyde (64 µl, 0.53 mmol) and $NaBH(AcO)_3$ (0.135 g, 0.63 mmol), AcOH (5 µl) in DCE (5 ml) dried with molecular sieves, was stirred under nitrogen at room temperature for 1 h. The solution was then washed with saturated aqueous $NaHCO_3$ (1 x 10 ml), water (1 x 10 ml) and brine (1 x 10 ml), dried with $MgSO_4$, and the solvent was removed under reduced pressure. The crude product was then purified by column chromatography on silica eluting with $CHCl_3$/EtOH (98:2). The product was isolated as a brown solid (0.097 g, 88%).

^{1}H NMR (500 MHz, aceton-*d*$_6$): δ 7.22 (td, J = 8, J = 1, 2H), 7.14 (d, J = 7, 2H), 6.99 (d, J = 8, 2H), 6.89-6.83 (m, 4H), 6.58 (d, J = 9, 2H), 4.58 (s, 4H), 4.27 (d, J = 7, 2H), 3.84 (s, 6H), 1.31 (d, J = 13, 18H).

^{31}P NMR (202.4 MHz, aceton-*d*$_6$): δ 53.9;

^{13}C NMR (125.7 MHz, aceton-*d*$_6$): δ 158.3, 151.2, 151.1, 145.1, 128.6, 127.8, 127.2, 121.1, 116.0, 111.1, 64.5, 64.0, 55.6, 50.8, 36.1, 35.6, 26.8;

MS (ES+): m/z (%) = 524.3 (100) [$M+H^+$];

HRMS (ES+): calcd for $C_{31}H_{43}NO_4P$ 524.2930, found 524.2928;

FT-IR (thin film): ν_{max} /cm^{-1} 2954, 2837, 1589, 1601, 1512;

m.p.: 141-142 °C.

Synthesis of 5 [3]

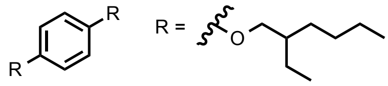

A mixture of benzene-1,4-diol (10 g, 91 mmol), 3-(bromomethyl)heptane (49 ml, 273 mmol) and K_2CO_3 (38 g, 273mmol) in a mixture of DMF (30 ml) and 2-butanone (200ml) was refluxed for 96 h. After cooling to room temperature, the solution was diluted with EtOAc (100 ml), washed with water (3 x 100 ml) and brine (1 x 100 ml), dried with $MgSO_4$, and the solvent was removed under reduced pressure. The crude material was isolated as a brown oil and used without further purification (26 g, 86%).

^{1}H NMR (400 MHz, $CDCl_3$): δ 6.88 (s, 4H), 3.84 (d, J = 6, 4H), 1.78-1.72 (m, 2H), 1.59-1.33 (m, 16H), 1.00-0.94 (m, 12H);
^{13}C NMR (100.6 MHz, $CDCl_3$): δ 153.6, 115.4, 71.1, 39.6, 30.7, 29.2, 24.0, 23.2, 14.2, 11.2;
MS (EI+): m/z (%) = 334 (70) [$M+H^+$];
HRMS (EI+): calcd for $C_{22}H_{38}O_2$ 334.287181, found 334.287220.

Synthesis of 6 [3]

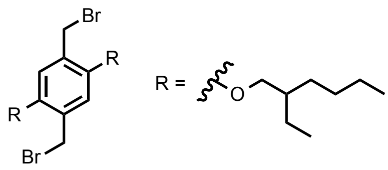

To a suspension of paraformaldehyde (2.3 g, 76 mmol) in acetic acid (250 ml) HBr (15 ml 31% in acetic acid) **5** (13 g, 38 mmol) was added and the mixture was heated to 70 °C and stirred for 12 h. After cooling to room temperature the mixture was poured in water (300 ml) and EtOAc (200ml) was added. The solution was washed with water (3 x 200 ml) then brine (1 x 100 ml), dried with $MgSO_4$ and removed under reduced pressure. The product, isolated as a brown oil was used without further purification (19.1 g, 96%).

^{1}H NMR (400 MHz, $CDCl_3$): δ 6.89 (s, 2H), 4.56 (s, 4H), 3.91 (d, J = 5, 4H), 1.816-1756 (m, 2H), 1.63-1.31 (m, 16H), 1.00-0.93 (m, 12H);
^{13}C NMR (100.6 MHz, $CDCl_3$): δ 150.7, 127.4, 114.2, 70.9, 39.7, 30.7, 29.2, 28.8, 24.1, 23.1, 14.2, 11.3;
MS (EI+): m/z (%) = 520 (35);
HRMS (EI+): calcd for $C_{24}H_{40}O_2 Br_2$ 518.13950, found 518.13886.

Synthesis of 7 [3]

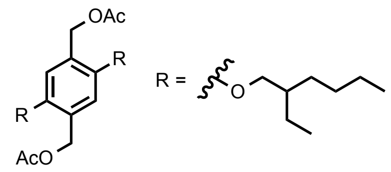

A solution of **6** (6.7 g, 13 mmol), KAcO (3.8 g, 38 mmol) and TBABr (0.6 g, 1.9 mmol) in a mixture of CH_3CN (165 ml) and $CHCl_3$ (85 ml) was refluxed for 24 h. The mixture was washed with water (2 x 200 mL) then brine (1 x 100 ml), dried with $MgSO_4$, and the solvent was removed under reduced pressure. The crude product (5.3 g, 85%) was used without further purification.

^{1}H NMR (400 MHz, $CDCl_3$): δ 6.89 (s, 2H), 5.14 (s, 4H), 3.85 (d, J = 5, 4H), 2.10 (s, 6H), 2.099-1.694 (m, 2H), 1.53-1.30 (m, 16H), 0.95-0.89 (m, 12H);
^{13}C NMR (100.6 MHz, $CDCl_3$): δ 170.9, 150.9, 125.0, 113.6, 71.0, 61.9, 39.6, 30.6, 29.2, 24.0, 23.1,14.1, 11.2;
MS (EI+): m/z (%) = 478 (50);
HRMS (EI+): calcd for $C_{28}H_{46}O_6$ 478.329440, found 478.328978.

Synthesis of 8 [3]

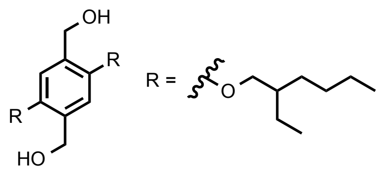

An aqueous solution of **7** (10 g, 20.0 mmol) and NaOH (4.2 g, 105 mmol) was refluxed for 12 h. The mixture was neutralized with HCl and extracted with DCM (3 x 50 ml). The combined organic layers were washed with brine (1 x 50 ml), dried with $MgSO_4$ and the solvent was removed under reduced pressure. The crude was purified by recrystallization from hexane and the product was isolated as a white solid (6.7 g, 85%).

^{1}H NMR (250 MHz, $CDCl_3$): δ 6.88 (s, 2H), 4.69 (s, 4H), 3.90 (d, J = 5, 4H), 1.77-1.69 (m, 2H), 1.55-1.29 (m, 16H), 0.98-0.89 (m, 12H);

^{13}C NMR (100.6 MHz, $CDCl_3$): δ 150.7, 129.0, 112.0, 70.9, 62.2, 39.6, 30.8, 29.1, 24.2, 23.1, 14.1, 11.2;

MS (EI+): m/z (%) = 394 (50);

HRMS (EI+): calcd for $C_{24}H_{42}O_4$ 394.308310, found 394.309132.

Synthesis of 9 [3]

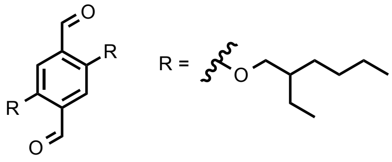

A mixture of **8** (0.5 g, 1.2 mmol), PCC (1.0 g, 4.6 mmol) in DCM (40 ml) was stirred for 4 h at room temperature. The reaction mixture was filtered through silica and the filtrated removed under reduced pressure. The pure product was obtained as a yellow oil (0.4 g, 81%).

^{1}H NMR (400 MHz, $CDCl_3$): δ 10.55 (s, 2H), 7.46 (s, 2H), 4.00 (d, J = 6, 4H), 1.83-1.72 (m, 2H), 1.56-1.32 (m, 16H), 0.98-0.91 (m, 12H);

^{13}C NMR (100.6 MHz, $CDCl_3$): δ 189.4, 155.4, 129.3, 111.5, 71.5, 39.4, 30.6, 29.1, 24.0, 23.0, 14.1, 11.2;

MS (ES+): m/z (%) = 391.3 (100) [$M+H^+$];

HRMS (ES+): calcd for $C_{24}H_{39}O_4$ 391.2848, found 391.2832;

FT-IR (thin film): ν_{max} /cm^{-1} 2959, 2929, 2861, 1682.

Synthesis of 10 [3]

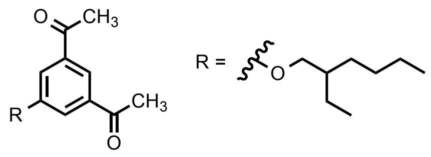

A mixture of dimethyl 5-hydroxyisophthalate (15 g, 71.0 mmol), 3-(bromomethyl)heptane (19 ml, 107 mmol) and K_2CO_3 (17 g, 123 mmol) in a mixture of DMF (30 ml) and 2-butanone (200ml) was refluxed for 72 h. After cooling to room temperature, the solution was diluted with EtOAc (100 ml), washed with water (3 x 100 mL) and brine (1 x 100 ml), dried with $MgSO_4$, and the solvent was removed under reduced pressure. The crude material was isolated as a clear yellow oil and used without further purification (18.7 g, 82%).

^{1}H NMR (400 MHz, $CDCl_3$): δ 8.26 (t, J = 1, 1H), 7.75 (d, J = 1, 2H), 3.94 (s, 6H), 3.92 (dd, J = 6, J = 1, 2H), 1.79-1.72 (m, 1H), 1.54-1.30 (m, 8H), 0.94-0.89 (m, 6H);
^{13}C NMR (100.6 MHz, $CDCl_3$): δ 166.3, 159.5, 131.7, 122.7, 119.8, 71.0, 52.4, 39.4, 30.5, 29.1, 23.9, 23.1, 14.1, 11.2;
MS (ES+): m/z (%) = 323.2 (20) [$M+H^+$], 340.2 (100) [M+Na];
HRMS (ES+): calcd for $C_{18}H_{27}O5$ 323.1858 found 323.1844;
FT-IR (thin film): ν_{max} /cm^{-1} 2957, 2930, 2874, 1728, 1595.

Synthesis of 11 [3]

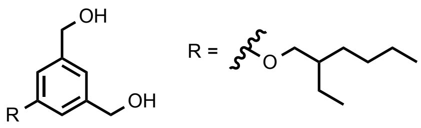

To a suspension of $LiAlH_4$ (4.4 g, 0.11 mol) in dry THF (100 ml) at 0 °C a solution of **10** (18 g, 0.05 mol) in THF (200 ml) was added dropwise over a period of 30 minutes. The reaction was stirred a room temperature for 2 h and then was quenched by addition of EtOAc (100 ml) at 0°C. The mixture was washed with water (2 x 100 mL) and brine (1 x 100 ml), dried with $MgSO_4$, and the solvent was removed under reduced pressure. The crude product (13.8 g, 94%) was used without further purification.

^{1}H NMR (400 MHz, $CDCl_3$): δ 6.84 (s, 1H), 6.77 (s, 2H), 4.55 (s, 4H), 3.82 (d, J = 6, 2H), 2.91 (s, 2H), 1.72-1.69 (m, 1H), 1.51-1.29 (m, 8H), 0.93-0.88 (m, 6H);
^{13}C NMR (100.6 MHz, $CDCl_3$): δ 159.7, 142.7, 117.3, 112.1, 70.6, 64.9, 39.4, 30.5, 29.1, 23.9, 23.1, 14.1, 11.1;
MS (ES+): m/z (%) = 267.2 (100) [$M+H^+$];
HRMS (ES+): calcd for 267.1960 $C_{16}H_{27}N_3P$ found 267.1968;
FT-IR (thin film): ν_{max} /cm^{-1} 3338, 3053, 2960, 2929, 2873, 1598.

Synthesis of 12 [3]

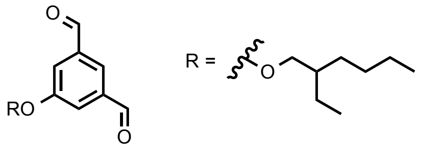

A mixture of **11** (8 g, 0.03 mol), PCC (25 g, 0.12 mol) in DCM (400 ml) was stirred for 2 h at room temperature. The reaction mixture was filtered through silica and the filtrate was evaporated under reduced pressure. The pure product was obtained as a clear yellow oil (7.2 g, 92%).

^{1}H NMR (250 MHz, $CDCl_3$): δ 10.04 (s, 2H), 7.93 (t, J = 1, 1H), 7.65 (d, J = 1, 2H), 3.95 (d, J = 6 2H), 1.82-1.72 (m, 1H), 1.52-1.28 (m, 8H), 0.96-0.87 (m, 6H);
^{13}C NMR (62.9 MHz, $CDCl_3$): δ 191.0, 160.6, 138.3, 124.0, 119.9, 71.3, 39.3, 30.5, 29.1, 23.8, 23.1, 11.1;
MS (ES+): m/z (%) = 263.2 (30) [M+H$^+$], 304.2 (100) [M+H$^+$ CH_3CN];
HRMS (ES+): calcd for $C_{16}H_{23}O_3$ 263.1647 found 263.1636;
FT-IR (thin film): ν_{max} /cm^{-1} 3054, 2959, 2930, 2859, 1702, 1593.

Synthesis of 13a

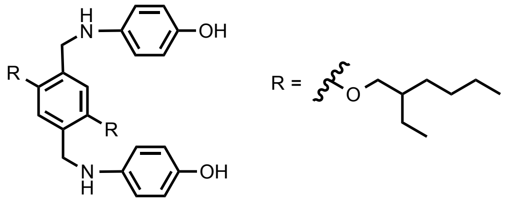

A mixture of **3a** (1.7 g, 15 mmol) **9** (2.5 g, 6.4 mmol) and $NaBH(AcO)_3$ (3.8 g, 16 mmol) in DCM (150 ml) was stirred under nitrogen at room temperature for 2 h. The solution was then washed with saturated aqueous $NaHCO_3$ (1 x 100 ml), water (1 x 100 ml) and brine (1 x 100 ml), dried with $MgSO_4$, and the solvent was removed under reduced pressure. The crude product was then purified by column chromatography on silica eluting with hexane/EtOAc (70:30). The product was isolated as a pink solid (2.7 g, 73%).

^{1}H NMR (400 MHz, $CDCl_3$): δ 6.87 (s, 2H), 6.68-6.58 (m, 8H), 4.24 (s, 4H), 3.81 (d, J = 5, 4H), 1.71-1.65 (m, 2H), 1.49-1.29 (m, 16H), 0.92-0.87 (m, 12H);

^{13}C NMR (101.2 MHz, $CDCl_3$): δ 150.8, 148.3, 142.2, 126.9, 116.1, 115.3, 113.1, 70.9, 45.4, 39.6, 30.7, 29.1, 24.1, 23.1, 14.1, 11.2;

MS (ES+): **m/z (%)** = 577.4005 (100) [$M+H^+$];

HRMS (ES+): calcd for $C_{36}H_{53}N_2O_4$ 577.4005, found 577.4005;

FT-IR (thin film): ν_{max} /cm^{-1} 3324, 2871, 2957,2927, 1513;

m.p.: 114-117 °C.

Synthesis of 14a

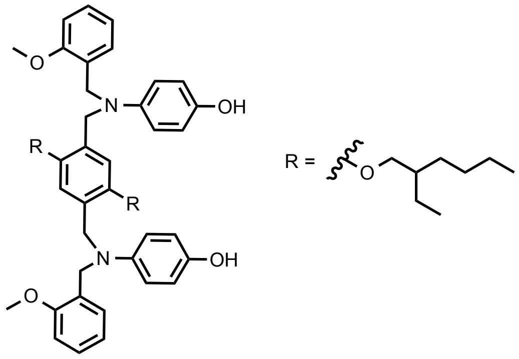

A mixture of 2-methoxybenzaldehyde (0.9 g, 6.9 mmol), of **13a** (1.0 g, 1.7 mmol) and $NaBH(AcO)_3$ (2.1 g, 9.7 mmol), AcOH (4 eq) in DCE (10 ml) dried with molecular sieves, was stirred under nitrogen at room temperature for 5 h. The solution was then washed with saturated aqueous $NaHCO_3$ (1 x 20 ml), water (1 x 20 ml) and brine (1 x 20 ml), dried with $MgSO_4$, and the solvent was removed under reduced pressure then purified by column chromatography on silica eluting with hexane/EtOAc (70:30). The product was isolated as a pink solid (1.1 g, 72%).

^{1}H NMR (400 MHz, CD_3CN): δ 7.21-7.12 (m, 4H), 6.92 (d, J = 8, 2H), 6.83 (t, J = 7, 2H), 6.74 (s, 2H), 6.60-6.51 (m, 8H), 6.08 (s, 2H), 4.50 (s, 4H), 4.48 (s, 4H), 3.80 (s, 6H), 3.64 (d, J = 5, 4H), 1.59-1.54 (m, 2H), 1.41-1.20 (m, 16H), 0.86-0.81 (m, 12H);
^{13}C NMR (100.6 MHz, CD_3CN): δ 157.4, 150.5, 148.2, 143.0, 127.8, 127.0, 126.4, 120.2, 115.7, 114.1, 111.9, 110.4, 70.9, 55.1, 50.6, 50.4, 39.3, 30.5, 28.9, 23.9, 22.9, 13.6, 10.7;
MS (ES+): m/z (%) = 817.5 (100) [$M+H^+$];
HRMS (ES+): calcd for $C_{52}H_{69}N_2O_6$ 817.5156, found 817.5161;
FT-IR (thin film): ν_{max} /cm^{-1} 3368, 2957, 2928, 2871, 1589, 1601, 1513;
m.p.: 121-124 °C.

Synthesis of 13b

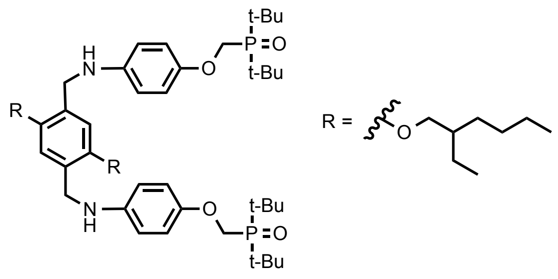

A mixture of **3b** (127 mg, 0.45 mmol), **9** (70.0 mg, 0.18 mmol, 1 eq) and $NaBH(AcO)_3$ (105 mg, 0.50 mmol) in DCE (5 ml) was stirred under nitrogen at room temperature for 2 h. The solution was then washed with saturated aqueous $NaHCO_3$ (1 x 10 ml), water (1 x 10 ml) and brine (1 x 10 ml), dried with $MgSO_4$, and the solvent was removed under reduced pressure. The crude product was then purified by column chromatography on silica eluting with EtOAc/MeOH (90:10). The product was isolated as a yellow solid (53.0 mg, 32%).

^{1}H NMR (400 MHz, MeOD): δ 6.91 (s, 2H), 6.81 (d, J = 9, 4H), 6.63 (d, J = 9, 4H), 4.36 (d, J = 7, 4H), 4.22 (s, 4H), 3.78 (d, J = 6, 4H), 1.64-1.59 (m, 2H), 1.41-1.28 (m, 52H), 0.89-0.85 (m, 12H);
^{31}P NMR (101.2 MHz, MeOD): δ 61.0;
^{13}C NMR (100.6 MHz, MeOD): δ 151.1, 151.0, 150.6, 143.5, 126.7, 114.9, 114.7, 112.3, 70.7, 63.0, 62.3, 43.5, 39.5, 35.2, 34.6, 30.5, 28.9, 25.3, 23.8, 22.7, 13.1, 10.3;
MS (ES+): **m/z (%)** = 925.6337 (100) [M+H$^+$];
HRMS (ES+): calcd for $C_{54}H_{91}N_2O_6P_2$ 925.6352, found 925.6337;
FT-IR (thin film): ν_{max} /cm^{-1} 3311, 2960, 2870, 1476, 1500, 1435, 1145.

Synthesis of 14b

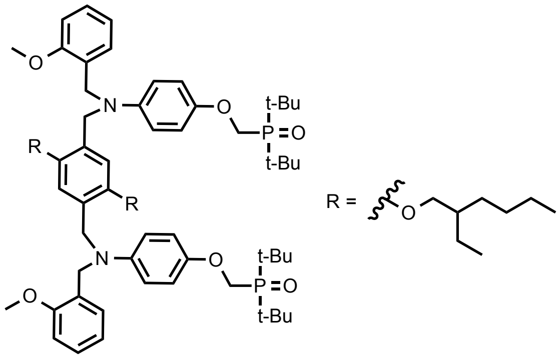

A mixture of 2-methoxybenzaldehyde (19 mg, 0.140 mmol), of **13b** (33 mg, 0.035 mmol) and NaBH(AcO)$_3$ (42 mg, 0.200 mmol), AcOH (4 eq) in DCE (250 µl) dried with molecular sieves, was stirred under nitrogen at room temperature for 7 h. The solution was then washed with saturated aqueous $NaHCO_3$ (1 x 10 ml), water (1 x 10 ml) and brine (1 x 10 ml), dried with $MgSO_4$, and the solvent was removed under reduced pressure then purified by column chromatography on silica eluting with $CHCl_3$/MeOH (97:3). The product was isolated as an orange oil (24 mg, 58%).

^{1}H NMR (500 MHz, CD_3CN): δ 7.20-7.17 (m, 2H), 7.09 (d, J = 7, 2H), 6.92 (d, J = 8, 2H), 6.82-6.77 (m, 6H), 6.71 (s, 2H), 6.58 (d, J = 9, 4H), 4.54 (s, 4H), 4.51 (s, 4H), 4.23 (d, J = 7, 4H), 3.77 (s, 6H), 3.63-3.62 (m, 4H), 1.56-1.51 (m, 2H), 1.35-1.18 (m, 52H), 0.831-0.775 (m, 12H);
^{31}P NMR (101.2 MHz, CD_3CN): δ 55.5;
^{13}C NMR (125.7 MHz, CD_3CN): δ 158.3, 151.3, 151.1, 151.1, 145.0, 128.8, 128.4, 127.5, 127.1, 121.1, 116.0, 114.4, 112.9, 111.4, 71.8, 64.3, 63.7, 55.9, 51.1, 40.0, 36.0, 35.6, 31.2, 29.7, 26.7, 24.6, 23.7, 14.4, 11.5;
MS (ES+): m/z (%) = 1165.7 (50) [M+H$^+$], 583.3 (100);
HRMS (ES+): calcd for $C_{70}H_{107}N_2O_8P_2$ 1165.7503, found 1165.7523;
FT-IR (thin film): ν_{max} /cm^{-1} 2955, 2926, 2872, 1688, 1601, 1500.

Synthesis of 15a

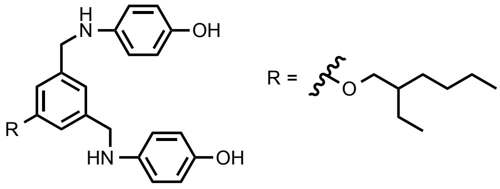

A mixture of **12** (1.6 g, 6.1 mmol) **3a** (1.7 g, 15 mmol) and NaBH(AcO)$_3$ (3.6 g, 16 mmol) in DCM (150 ml) was stirred under nitrogen at room temperature for 2 h. The solution was the washed with saturated aqueous NaHCO$_3$ (1 x 100 ml), water (1 x 100 ml) and brine (1 x 100 ml), dried with MgSO$_4$ and the solvent was removed under reduced pressure. The crude product was then purified by column chromatography on silica eluting with hexane /EtOAc (50:50). The product was isolated as a pink solid (0.53 g, 20%).

^{1}H NMR (500 MHz, CD$_3$CN): δ 6.92 (s, 1H), 6.79 (s, 2H), 6.62-6.60 (m, 4H), 6.50-6.48 (m, 4H), 4.15 (s, 4H), 3.81 (d, J = 6, 2H), 1.68-164 (m, 1H), 1.49-1.29 (m, 8H), 0.90 (t, J = 7, 6H);

^{13}C NMR (125.7 MHz, CD$_3$CN): δ 160.7, 149.5, 143.2, 143.1, 119.6, 116.8, 115.3, 112.9, 71.2, 49.3, 40.2, 31.3, 29.8, 24.6, 23.8, 14.5, 11.5;

MS (ES+): m/z (%) = 449.3 (100) [M+H$^+$];

HRMS (ES+): calcd for $C_{28}H_{37}N_2O_3$ 449.2804 found 449.2785;

FT-IR (thin film): ν_{max} /cm^{-1} 3586, 3395, 3054, 2961, 2930, 2873, 1596, 1515.

m.p.: 132-136 °C

Synthesis of 16a

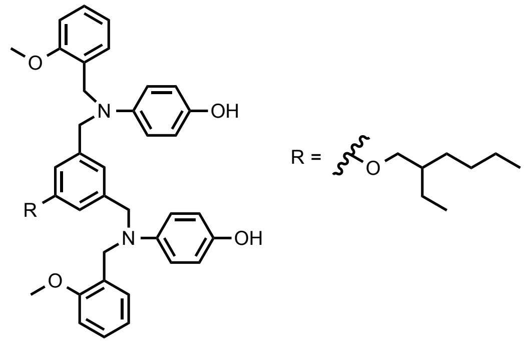

A mixture of 2-methoxybenzaldehyde (0.13 g, 0.97 mmol), of **15a** (0.11 g, 0.24 mmol) and $NaBH(AcO)_3$ (0.29 g, 1.4 mmol), AcOH (4 eq) in DCE (1 ml) dried with molecular sieves, was stirred under nitrogen at room temperature for 2 h. The solution was then washed with saturated aqueous $NaHCO_3$ (1 x 10 ml), water (1 x 10 ml) and brine (1 x 10 ml), dried with $MgSO_4$ and the solvent was removed under reduced pressure. The crude product was then purified by column chromatograph on silica eluting with hexane/EtOAc (70:30). The product was isolated as a red oil (0.11 g, 66%).

^{1}H NMR (500 MHz, CD_3CN): δ 7.18 (td, J = 8, J= 1, 2H), 7.10 (dd, J = 7, J = 1, 2H), 6.91 (d, J = 8, 2H), 6.83-6.80 (m, 2H), 6.75 (s, 1H), 6.63 (s, 2H), 6.57-6.50 (m, 8H), 4.42 (s, 4H), 4.41 (s, 4H), 3.79 (s, 6H), 3.70 (d, J = 6, 2H), 1.62-1.57 (m, 1H), 1.43-1.20 (m, 8H), 0.88-0.83 (m, 6H).

^{13}C NMR (125.7 MHz, CD_3CN): δ 160.8, 158.5, 149.4, 143.8, 142.8, 129.0, 128.9, 127.9, 121.2, 118.9, 116.6, 115.7, 112.4, 111.5, 71.3, 56.6, 56.0, 51.4, 40.2, 31.3, 29.9, 24.7, 23.9, 14.5, 11.5;

MS (ES+): m/z (%) = 689.4 (100) [$M+H^+$];

HRMS (ES+): calcd for $C_{44}H_{53}N_2O_5$ 689.3954 found 689.3922;

FT-IR (thin film): ν_{max} /cm^{-1} 3370, 2957, 2929, 2874, 1597.

Synthesis of 15b

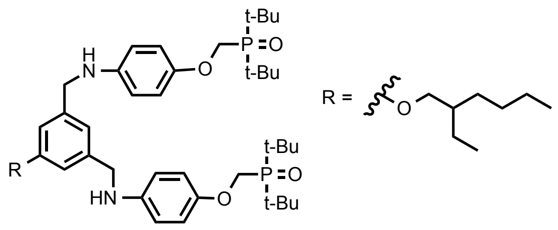

A mixture of **12** (32 mg, 0.12 mmol) and **3b** (87 mg, 0.31 mmol) mmol) in $CHCl_3$ (5 ml) was stirred under nitrogen at room temperature for 48 h. The solvent was then removed under reduced pressure, the crude dissolved in MeOH. $NaBH_4$ (28 mg, 0.72 mmol) was added at 0°C and the solution left stirring for 10 min. The solution was neutralized with HCl 2M and extracted with DCM (3x10 ml). The organic layers were combined and washed brine (1 x 10 ml). The solution was dried with $MgSO_4$ and the solvent was removed under reduced pressure. The crude material was then purified by column chromatograph on silica eluting with EtOAc/MeOH (90:10). The product was isolated as a yellow oil (40 mg, 41%).

^{1}H NMR (400 MHz, CD_3CN): δ 6.92 (s, 1H), 6.80-6.76 (m, 6H), 6.57-6.53 (m, 4H), 4.26 (d, J = 6, 4H), 4.19 (s, 4H), 3.80 (d, J = 6, 2H), 1.67-1.61 (m, 1H), 1.46-1.27 (m, 44H), 0.92-0.84 (m, 6H).

^{31}P NMR (162.0 MHz, CD_3CN): δ 55.37;

^{13}C NMR (100.6 MHz, CD_3CN): δ 159.7, 150.8, 150.7, 143.7, 142.3, 118.4, 115.3, 113.7, 111.7, 70.3, 63.6, 62.9, 47.8, 39.1, 35.2, 34.6, 30.3, 28.8, 25.8, 23.6, 22.8, 13.4, 10.4;

MS (ES+): m/z (%) = 797.5 (100) [M+H+];

HRMS (ES+): calcd for $C_{46}H_{75}N_2O_5P_2$ 797.5151 found 797.5120;

FT-IR (thin film): ν_{max} /cm^{-1} 3229, 2958, 1595, 1510.

Synthesis of 16b

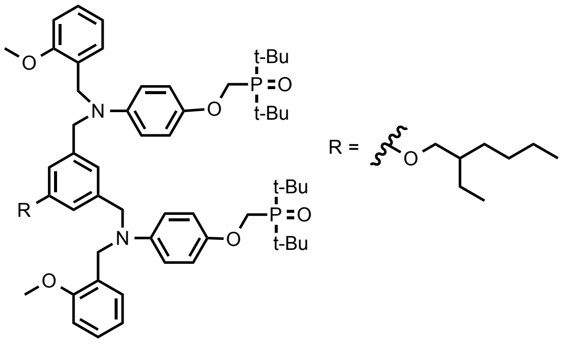

A mixture of 2-methoxybenzaldehyde (10 mg, 0.071 mmol), of **15b** (14 mg, 0.017 mmol) and NaBH(AcO)$_3$ (20 mg, 0.094 mmol), AcOH (4 eq) in DCE (100 μl) dried with molecular sieves, was stirred under nitrogen at room temperature for 12 h. The solution was then washed with saturated aqueous $NaHCO_3$ (1 x 10 ml), water (1 x 10 ml) and brine (1 x 10 ml), dried with $MgSO_4$ and the solvent was removed under reduced pressure. The crude product was then purified by column chromatograph on silica eluting with EtOAc/MeOH (97:3). The product was isolated as an orange oil (10.2 mg, 57%).

^{1}H NMR (500 MHz, CD_3CN): δ 7.20 (td, J = 8, J = 1, 2H), 7.07 (dd, J = 7, J = 1, 2H), 6.94 (d, J = 8, 2H), 6.83 (d, J = 7, 2H), 6.78-6.76 (m, 4H), 6.74 (s, 1H), 6.62 (s, 2H), 6.56 (d, J = 9, 4H), 4.49 (s, 4H), 4.45 (s, 4H), 4.24 (d, J = 6, 4H), 3.80 (s, 6H), 3.72 (d, J = 6, 2H), 1.62-1.57 (m, 1H), 1.41-1.25 (m, 44 H), 0.87-0.83 (m, 6H);
^{31}P NMR (202.4 MHz, CD_3CN): δ 55.4;
^{13}C NMR (125.7 MHz, CD_3CN): δ 160.8, 158.4, 151.4, 151.3, 145.0, 142.5, 128.9, 128.5, 127.5, 121.2, 118.5, 116.1, 114.8, 112.3, 111.5, 71.2, 64.4, 63.8, 56.1, 51.2, 40.1, 36.1, 35.6, 31.2, 29.7, 26.8, 23.8, 14.4, 11.4;
MS (ES+): m/z (%) = 1037.6 (100) [M+H$^+$];
HRMS (ES+): calcd for $C_{62}H_{91}N_2O_7P_2$ 1037.6302 found 1037.6295;
FT-IR (thin film): ν_{max} /cm^{-1} 3048, 2871, 2959, 2916, 2848, 1597, 1512.

NMR Binding Studies

All binding constants were measured by means of NMR titrations. A known concentration of host solution (0.5-1 mM) in deuterated toluene or chloroform was prepared. A fraction of the host stock solution (0.45-0.6 ml) was transferred to a NMR tube. The guest solution (1-100 mM) was then prepared by dissolving it in the host stock solution. In this way the concentration of host is maintained constant throughout the titration. ^{1}H NMR and ^{31}P NMR spectra were recorded after successive additions of aliquots of guest solution. The observed changes in chemical shift were analysed using a purpose-written fitting program in Microsoft Excel. Errors are quoted as two times the standard deviation.

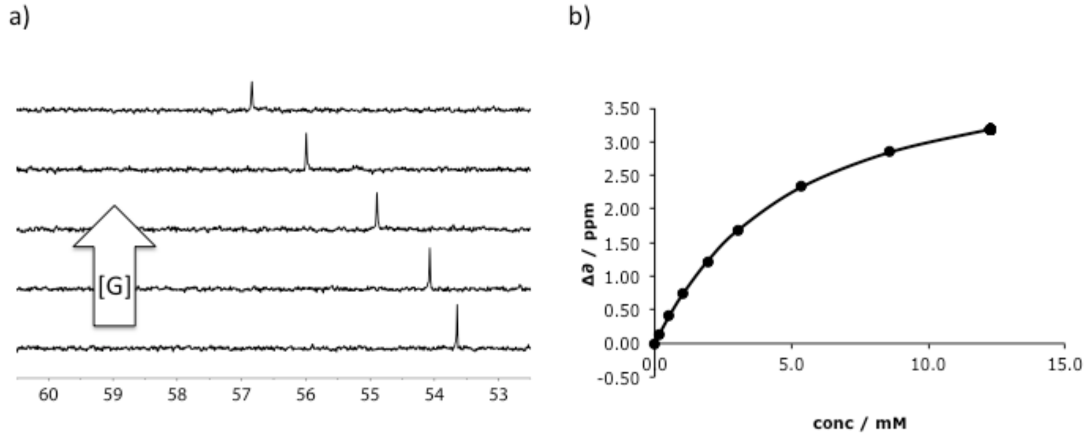

Figure S1 ^{31}P NMR chemical shift as a function of guest concentration for addition of 4a to 4b. The line represents the best fit to a 1:1 binding isotherm; d) 162 MHz ^{31}P NMR titration data for addition of 4a to 4b in toluene-*d*8 at 298 K.

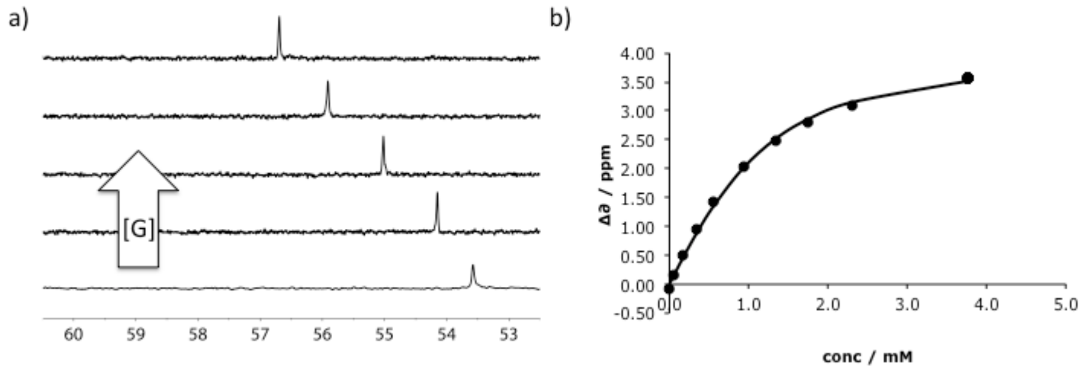

Figure S2 ^{31}P NMR chemical shift as a function of guest concentration for addition of 14a to 14b. The line represents the best fit to a 1:1 binding isotherm; d) 162 MHz ^{31}P NMR titration data for addition of 14a to 14b. in toluene-*d*8 at 298 K.

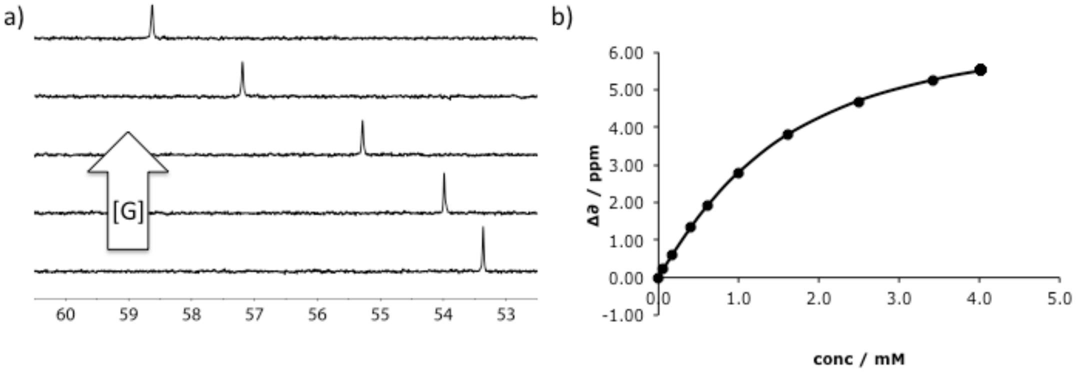

Figure S3 a) ^{31}P NMR chemical shift as a function of guest concentration for addition of 16a to 16b. The line represents the best fit to a 1:1 binding isotherm; b) 162 MHz ^{31}P NMR titration data for addition of 16a to 16b in toluene-*d*8 at 298 K.

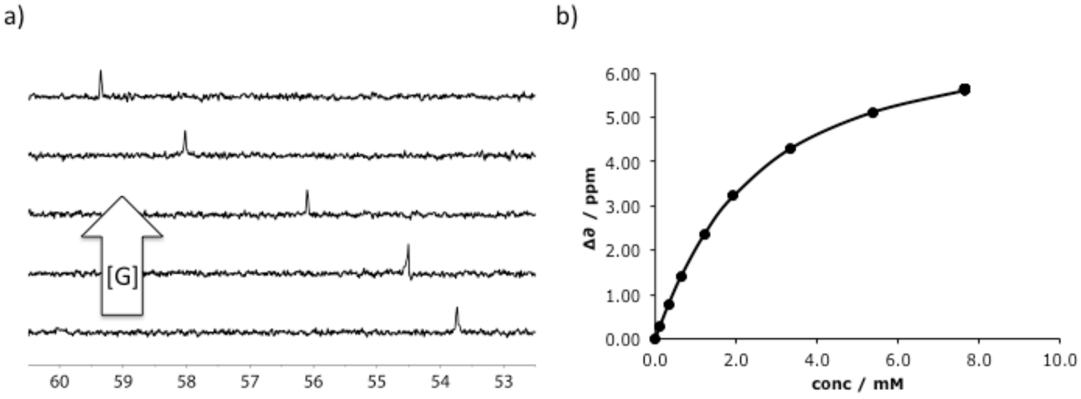

Figure S4 a) ^{31}P NMR chemical shift as a function of guest concentration for addition of 16a to 14b. The line represents the best fit to a 1:1 binding isotherm; b) 162 MHz ^{31}P NMR titration data for addition of 16a to 14b in toluene-*d*8 at 298 K.

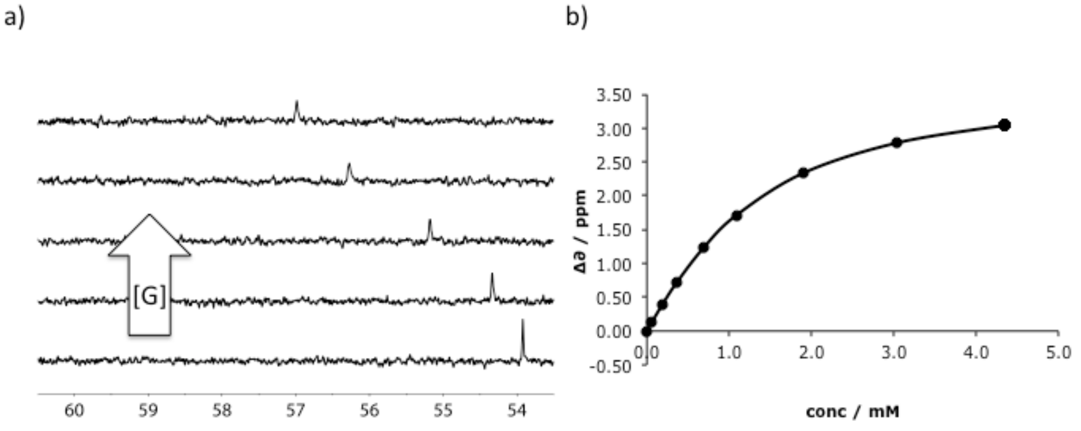

Figure S5 a) ^{31}P NMR chemical shift as a function of guest concentration for addition of 14a to 18b. The line represents the best fit to a 1:1 binding isotherm; b) 162 MHz ^{31}P NMR titration data for addition of 14a to 18b in toluene-*d*8 at 298 K.

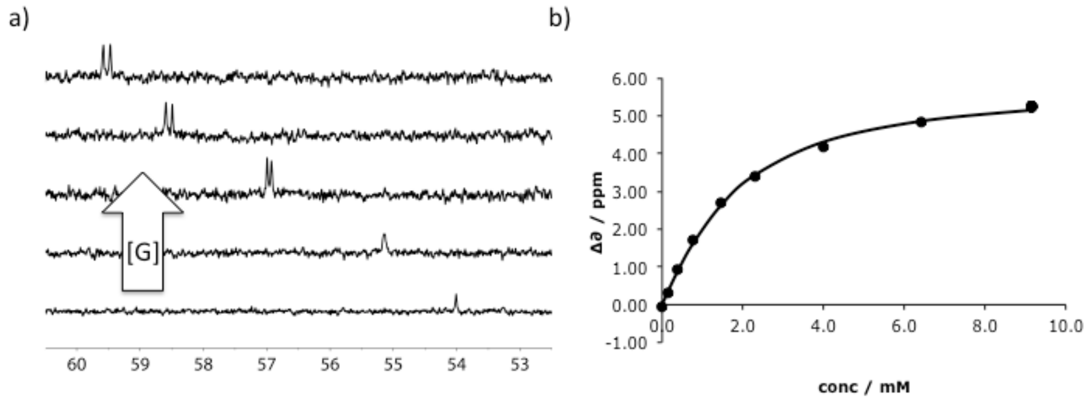

Figure S6 a) ^{31}P NMR chemical shift as a function of guest concentration for addition of 16a to 14b. The line represents the best fit to a 1:1 binding isotherm; b) 162 MHz ^{31}P NMR titration data for addition of 16a to 18b in toluene-*d*8 at 298 K.

References

1 Raeppel, S; Raeppel, F; Suffert, J. *Synlett* 1998, 794.

2 (a) A. F. Abdel-Magid and S. J. Mehrman, *Org. Process Res. Dev.*, 2006, **10**, 971; (b) A. F. Abdel-Magid, K. G. Carson, B. D. Harris, C. A. Maryanoff and R. D. Shah, *J. Org. Chem.*, 1996, **61**, 3849.

3 Wang, B; Wasielewski, M. R. *J. Am. Chem. Soc.* 1997, **119**, 12-21.
